# Controlling a motorized orthosis to follow elbow volitional movement: tests with individuals with pathological tremor

**DOI:** 10.1186/s12984-019-0484-1

**Published:** 2019-02-01

**Authors:** Gil Herrnstadt, Martin J. McKeown, Carlo Menon

**Affiliations:** 1 Menrva Research Group, Schools of Mechatronic Systems Engineering and Engineering Science, Simon Fraser University, Burnaby, Canada; 2 Department of Medicine (Neurology) and Pacific Parkinson’s Research Centre, University of British Columbia, Vancouver, Canada

**Keywords:** Pathological tremor, Orthosis, Assistive robot, Tremor suppression, Control

## Abstract

**Background:**

There is a need for alternative treatment options for tremor patients who do not respond well to medications or surgery, either due to side effects or poor efficacy, or that are excluded from surgery. The study aims to evaluate feasibility of a voluntary-driven, speed-controlled tremor rejection approach with individuals with pathological tremor. The suppression approach was investigated using a robotic orthosis for suppression of elbow tremor. Importantly, the study emphasizes the performance in relation to the voluntary motion.

**Methods:**

Nine participants with either Essential Tremor (ET) or Parkinson’s disease (PD) were recruited and tested off medication. The participants performed computerized pursuit tracking tasks following a sinusoid and a random target, both with and without the suppressive orthosis. The impact of the Tremor Suppression Orthosis (TSO) at the tremor and voluntary frequencies was determined by the relative power change calculated from the Power Spectral Density (PSD). Voluntary motion was, in addition, assessed by position and velocity tracking errors.

**Results:**

The suppressive orthosis resulted in a 94.4% mean power reduction of the tremor (*p* < 0.001) – a substantial improvement over reports in the literature. As for the impact to the voluntary motion, paired difference tests revealed no statistical effect of the TSO on the relative power change (*p* = 0.346) and velocity tracking error (*p* = 0.283). A marginal effect was observed for the position tracking error (*p* = 0.05). The interaction torque with the robotic orthosis was small (0.62 Nm) when compared to the maximum voluntary torque that can be exerted by adult individuals at the elbow joint.

**Conclusions:**

Two key contributions of this work are first, a recently proposed approach is evaluated with individuals with tremor demonstrating high levels of tremor suppression; second, the impact of the approach to the voluntary motion is analyzed comprehensively, showing limited inhibition. This study also seeks to address a gap in studies with individuals with tremor where the impact of engineering solutions on voluntary motion is unreported. This study demonstrates feasibility of the wearable technology as an effective treatment that removes tremor with limited impediment to intentional motion. The goal for such wearable technology is to help individuals with pathological tremor regain independence in activities affected by the tremor condition. Further investigations are needed to validate the technology.

## Introduction

Over ten subtypes of disorders associated with pathological tremor have been identified by the medical community [1] of which Essential Tremor (ET) and Parkinson’s Disease (PD) are considered the most pervasive. Overall pathological tremor prevalence ranges from 2% to well over 10% in the elderly (65 years or older) [2–4]. A large percentage, some estimates are as high as 60%, of those affected by tremor experience disability in their activities of daily living [5, 6], and more than a quarter struggle to find relief through conventional treatments [7]. Treatment with pharmacotherapy can be challenging as individual responses vary widely; a typical scenario is that a given medication is partially efficacious at low dosages, but increasing dosage results in a trade-off between efficacy and associated side effects [8]. When medications are effective, the expected tremor reduction is around 50–60% [6, 9]. Individuals with a disabling or medication refractory tremor, may have the option for one of several surgical procedures in the form of Deep Brain Stimulation (DBS), lesioning techniques such as γ knife radiosurgery (invasive), and magnetic resonance-guided focused ultrasound (non-invasive) [9, 10]. In practice, less than 2% of PD patients are targeted for treatment with DBS [11], with about 40,000 PD and ET patients undergoing DBS worldwide by 2013 [12]. A small but considerable proportion of individuals undergoing DBS experience side effects or complications. About 70–90% of DBS patients experience reduction of tremor that can range from 60 to 90% [8, 13]. Focused ultrasound offers a non-invasive thalamotomy procedure that has been demonstrated to reduce tremor [14]. Risks for surgical procedures include intracerebral hemorrhage and other potential neurologic impairments [15].

There is, therefore, a persuasive case for alternative therapies for individuals with pathological tremor who respond poorly to medications and DBS or for whom surgery is not an option. Perhaps one of the strongest arguments for robotic devices for treatment of tremor is that such devices could successfully treat tremors arising from different biological etiologies (e.g. resting, postural and kinetic) with possible minor tremor-specific optimizations.

A number of systems designed for the suppression of upper limb tremor employed suppressive technologies such as viscous and magnetic fluids, magnetic particle brakes, pneumatic actuators and DC motors [16–26] while others employed Functional Electrical Stimulation (FES) [4, 27–35]. Of the above systems, several were demonstrated with individuals with tremor [17–20, 27–32] resulting in ~ 20–88% tremor attenuation levels, although attenuation levels were not computed consistently across the studies and should therefore be considered cautiously. The remaining systems focused on design or experimental testing whereby the human motion (tremor and voluntary) was simulated physically or in software [4, 16, 22–26, 33–35]. Limitations of non FES technologies tend to be related to size and weight whereas the main limitations associated with FES are muscle fatigue, stimulation discomfort and difficulty in accessing specific muscles through surface electrodes. All technologies may require some amount of customized tuning per individual’s physiology, however, potentially more so with FES.

Common tremor suppression methods involve estimating the tremor and applying an opposite canceling signal, for example in the form of velocity or force exerted by an actuator. In the case of FES an out of phase stimulation may be applied to, say, the flexor muscle concurrent with its antagonist tremor burst. Alternatively, some approaches modulate the impedance of the human-machine system [20, 24, 29]. In the case of FES this may involve stimulation of both flexor and extensor simultaneously in order to increase the stiffness and viscosity of the limb. Another recent approach for FES involves stimulating below the motor threshold to mitigate the issue of fatigue [4, 28]. The results provide some evidence of residual suppression even after stimulation has stopped, however, with larger variability in performance and lower tremor attenuation.

When mechanically suppressing tremor, there is a risk of preventing the individual with tremor from performing volitional movements. Notably, the potential negative effects on the volitional movement are seldom addressed in the literature. Rocon et al. employed a feedback loop aimed at reducing the forces resisting the voluntary movement [20], however, with reported results focusing on the tremor motion. A study by Taheri et al. reported the actuator resistive forces to the voluntary motions in a tremor simulation system [26]. One study involving a single individual exhibiting intention tremor, due to MS, was identified that reported the impact of FES suppression on the voluntary motion in a step-target tracking task [32]. No other studies with individuals with tremor were identified that quantitively assess the impact on the volitional motion using an engineering solution.

Different from conventional suppression techniques, the orthosis employed in this work tracks the voluntary motion, estimated from a force signal [36], while the tremor signal is seen as interference to the volitional motion and is consequently rejected by the controller.

The aim of this work is to show viability of the suppression approach, using an elbow orthosis prototype [37], tested with individuals with pathological tremor. The above aim can be explored as two separate questions. First, is the approach effective in suppressing the involuntary motion, and second, is the interference to the voluntary motion quantifiably limited. The application of the suppression approach with an orthosis can provide an important therapeutic alternative for individuals with tremor, with potential to improve independence and quality of life.

## Methods

### Participant selection

Nine participants were recruited to evaluate the tremor suppression orthosis. Individuals who have been diagnosed with mild to severe tremor were considered for the study. The following conditions were excluded: previous surgical operation or injuries to the arm, non-tremor related arm disability, and previous surgical intervention to treat tremor or a neurologic condition other than pathological tremor that affects the arms. Participants were recruited through the International Essential Tremor Foundation and the Movement Disorder Clinic, University of British Columbia Hospital. Participants were invited for up to two sessions. Each session lasted approximately 2 h. Additionally, participants were asked to abstain from taking medications 12 h prior to the study and advised to refrain from drinking alcohol 24 h before the study. A tremor severity assessment was carried out at the end of each study session using the performance section of The Essential Tremor Rating Assessment Scale (TETRAS) [38, 39]. The participants’ details are provided in Table 1.

### Suppression approach and elbow orthosis

In the voluntary-driven suppression approach, the users’ voluntary torque was used to guide the orthosis such that it tracked the voluntary motion with little resistance and thus perceived as quasi-transparent by the user, while the tremor component was considered a disturbance and was rejected. It should be noted that a fully transparent device, in the context of human-robot interaction, is one that induces no forces on the user [40]. The implementation was as follows: the forces between the user and the mechanical suppression system were recorded with a force transducer. The forces were then filtered to isolate the voluntary component, which was then converted to a velocity signal, representing the voluntary motion, and passed to the outer loop admittance controller. A closed loop internal velocity controller guaranteed the tracking of the aforementioned velocity while rejecting the tremor, considered a disturbance. The admittance and speed controllers adopted a Proportional-Integral-Derivative (PID) and Proportional-Integral structures, respectively. The suggested suppression approach and control strategy have been recently demonstrated using a preliminary engineered system [36]. Subsequently, an elbow Tremor Suppression Orthosis (TSO) prototype was developed and experimentally tested with the suppression approach [37] while the human input was robotically simulated. The previously developed TSO is employed in this study with individuals with tremor to show feasibility of the suppression approach. Modifications to the suppression approach controller relative to previous work, are reviewed in section Updates to control system.

**Fig. 1:**
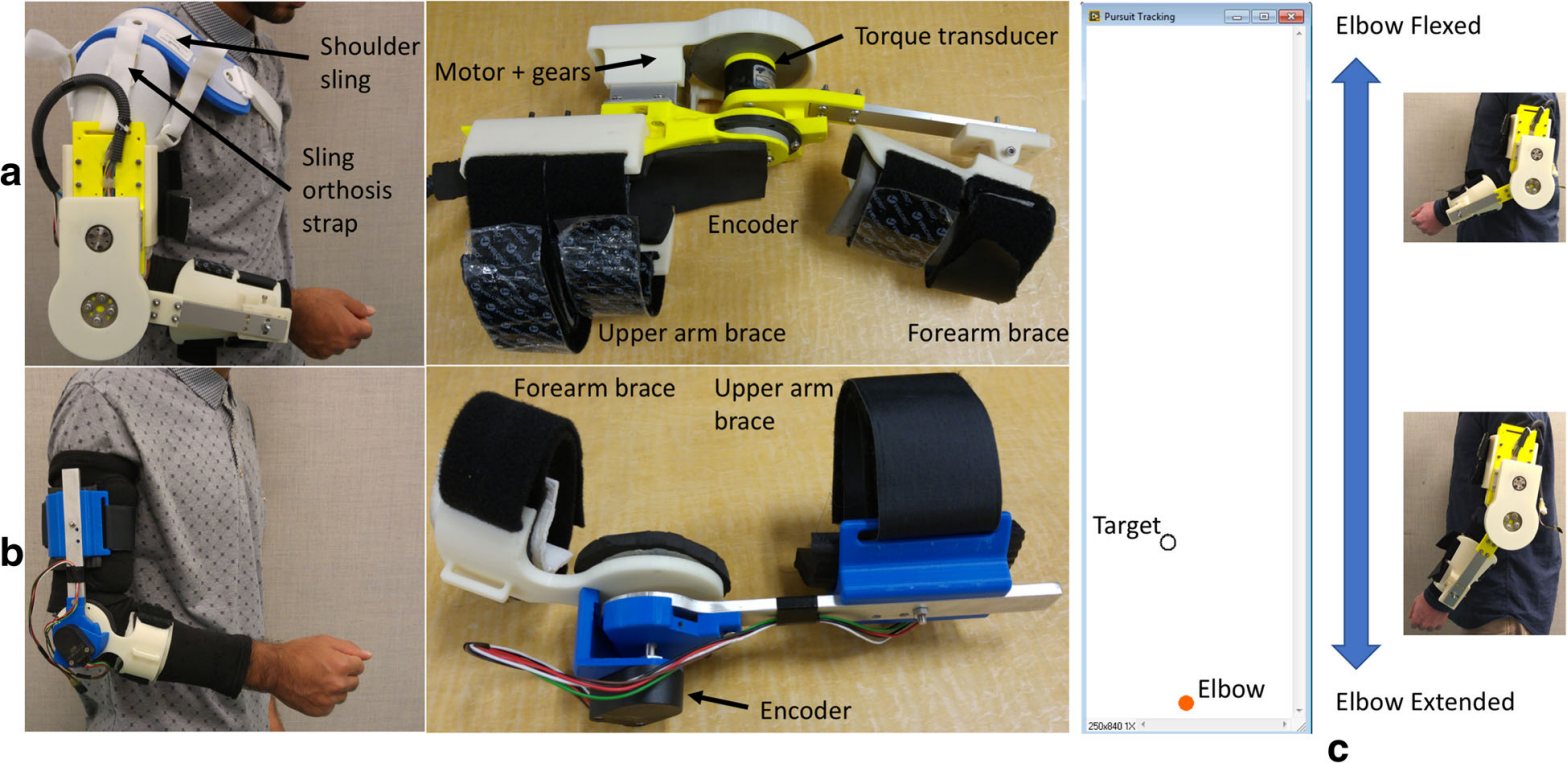
Orthoses and Computer Interface. **a** TSO components and TSO donned. **b** MO components and MO donned. **c** Pursuit target graphical interface seen by the study participants. Cursors moved vertically. The outlined circle indicates the position of the target on the screen and the filled orange circle indicates the participant’s elbow cursor position. Fully extended elbow position was considered as the zero angle and corresponded to the cursor located at the bottom of the graphical interface window

The TSO (Fig. 1a) is actuated with a brushless DC motor, connected to a commercial spur gearbox (26:1) in combination with a custom gear reduction (120:72), is embedded with a torque sensor and an encoder and weighs 1700 g. A shoulder sling was used (AliMed® Hemi Shoulder Sling) to help keep the TSO in place during the study and distribute its weight. Additionally, a Measurement Orthosis (MO) was developed in order to measure the free, unobstructed motion (containing the tremor) exhibited by the study participants. Attempting to measure the free motion with the TSO, would result in significant impedance due to the motor, gears and added mass. The MO, weighing 300 g, was composed of a lightweight brace and an encoder (Fig. 1b). Both orthoses are predominantly 3D fabricated with a thermoplastic polymer (ABS), and connect with straps at the upper arm brace and forearm brace. Aluminum bars are used for the TSO forearm link and the MO upper arm link. The weight of the MO forearm brace including padding was 42.5 g, similar to that of a wrist watch. Control and measurement of the devices was implemented in NI LabVIEW 2014.

### Updates to control system

Several changes relative to the previous control approach were implemented in this work. First, an Adaptive Band Pass Filter (ABPF) was implemented to perform online separation of the measured torque into its voluntary and tremor components [41], whereas previously a Kalman Filter was used. The ABPF essentially involves a band pass filter with a center frequency that is feedback adaptable. The ABPF was first initialized offline with the tremor center (fundamental) frequency. The ABPF could adapt about the center frequency (±1.5 Hz), based on an online frequency estimation. The tremor component extracted with the ABPF is then subtracted from the total torque signal to obtain the voluntary component estimation. The ABPF frequency step parameter and filter transfer function parameter were *∆f* = 0.25 *Hz*, and *β* = 1, respectively. In addition, a gain of 0.8 was used with the Band Pass Filter. *f*_*mod*_, the center tremor frequency, was calculated individually for each participant as described in section Experimental protocol. A ~ 9 Hz noise component, likely related to the actuation system, remained in the voluntary torque signal after subtraction of the ABPF signal and was consequently filtered with a first order Low Pass Filter (LPF) with a cut-off frequency of 5 Hz. The 5 Hz cut off frequency limited voluntary signal phase shift distortion. Second, based on the system response in the authors’ previous work, a need for additional damping was identified [36]. Consequently, a non-linear integral correction factor, utilizing a velocity feedback, was added to the admittance controller as follows

$$ {I}_{cf}=\frac{1}{1+10\left(\raisebox{1ex}{${\left(1-{v}_n\right)}^2$}\!\left/ \!\raisebox{-1ex}{${V}^2$}\right.\right)} $$

1 $$ {v}_n=\raisebox{1ex}{$\left|{v}_m\right|$}\!\left/ \!\raisebox{-1ex}{$V$}\right.,{v}_n\le 1 $$

where *v*_*m*_, *v*_*n*_ and *V* are the measured velocity, the normalized velocity and the full scale velocity parameter, selected as 2 rad/s. The resultant expression in (1) is *I*_*cf*_ = 8/(5(|*v*_*m*_| − 2)^2^ + 8). The controller integral gain was then scaled by *I*_*cf*_. The integral correction factor values ranged approximately between 0.28 ≤ *I*_*cf*_ ≤ 1. At high velocities, the integral gain remained unchanged (*I*_*cf*_ = 1), while for slow velocities it was reduced, resulting in a more damp response. It should be noted, the concept of the integral correction factor is similar to that of gain scheduling. Third, the state feedback used in [36] was omitted from the controller to reduce tuning complexity. Furthermore, its contribution to the system performance was considered limited. Lastly, the software sampling rate was increased to 100 Hz.

### Experimental protocol

The experimental protocol involved pursuit tracking tasks where the participants viewed a target moving on a computer screen (outlined circle in Fig. 1c) and were asked to follow the target cursor, i.e. match its current position and velocity, by flexing and extending their elbow. The participants’ elbow joint movement was translated to a cursor movement on the screen (filled orange circle in Fig. 1c). Since only the elbow joint movements were measured and suppressed, subjects were instructed to try and relax adjacent shoulder and hand joints, keeping their upper arm alongside their body as demonstrated in Fig. 1c, while performing flexion and extension movements of the elbow. Protocol tasks were repeated with both the MO and the TSO. One of two scaling factors (0.218 or 0.246 cm/deg) was used to match the elbow cursor with the target cursor motion range, depending on the individual’s Range Of Motion (ROM). Participants’ ROM was assessed with the MO by reading the encoder output angle while participants were asked to flex and extend their forearm through the full range. Two types of target profiles were used, namely a sinusoid and a pseudo-random ramp shape profile, each implemented with slow and fast velocities. The sinusoid target profiles were defined as 75.4 sin(0.6*πt*) deg./s for the slow case, and 125.5 sin(*πt*) deg./s for the fast case. The above resulted in an elbow ROM of about 100 deg. The pseudo-random target profiles were defined by successive random position targets between 0 and 100 deg., to which a ramp shape velocity profile was fitted. The ramp profile top velocities were 22.2 and 50 deg./s for the slow and fast cases, respectively, while the acceleration/deceleration were set to 27.75 and 83.3 deg./s^2^ for the slow and fast cases, respectively. The continuous target position associated with the random target velocity ramp profile followed a parabolic shape. All the above target profile parameters are for the larger scaling factor (0.246 cm/deg).

Pursuit tracking tasks have the added value of providing the desired movement (the target) relative to which the participants’ actual motion can be analysed. A summary of the experimental protocol and tasks sequence is available in Table 1. Identical tasks and target profiles were used for all participants. Up to two training repetitions were offered for each motion profile with either device. However, study participants had no prior exposure to the pseudo-random profiles used. All participants, except T08, were tested on their more severe side. The adaptation of the TSO to either the left or right arm is straightforward and was done ahead of the testing session.

**Table 1:** Participant data

Participant	Gender	Age	Duration (y)	Handedness	Severe Side	TETRAS	Diagnosis
T01	F	59	55	R	L	27.5	ET
T02	M	65	5	R	R	21	PD (tremor dominant)
T03	M	66	3	R	R	22.5	PD (also ET)
T04	M	69	4	R	R	24	ET
T05	M	56	53	R	R	26.5	ET
T06	M	71	10	R	R	16	ET
T07	M	69	3	R	L	21	N/A
T08	F	81	20	R	L	24.5	ET
T09	F	63	6	R	R	8	ET
Mean	–	66.6 (7.7)	17.7 (21.3)	–	–	21.2 (6)	–

Duration refers to disease duration; disease duration, handedness and more severe side were determined based on self-report and/or a neurologist assessment. The TETRAS performance section scale is scored out of a total of 64

As mentioned in section Updates to control system, the ABPF filter implementation involved selecting a center tremor frequency *f*_*mod*_. To this end, the following protocol steps were performed:

1. The MO was donned and the protocol tasks executed (see Table 1).
2. The 1st harmonic tremor frequency was extracted from the MO tasks data. The extraction calculation is explained in section Spectral analysis.
3. The calculated 1st harmonic frequency was fed to the ABPF algorithm as *f*_*mod*_.
4. The TSO was donned and the protocol tasks from Table 1 were executed again.

### Data processing and expected outcomes

The MO was incorporated in the protocol in order to record the free motion and serve as a reference for the TSO. The performance of the TSO was always compared to the MO in both the spectral and time domains. Since the MO measured the free tremor, it was also useful in identifying the users’ typical tremor center frequency for initialization of the ABPF, as indicated in section Experimental protocol. Although the MO may introduce some motion attenuation through its joint friction and forearm brace inertia, it was considered negligible resulting in approximately zero attenuation. Position and velocity signals were available directly from the embedded encoders in the MO and TSO. Torque measurement was available from the TSO torque sensor.

#### Spectral analysis

The main analysis tool for the spectral domain involved the Power Spectral Density (PSD). The PSD describes the power in the signal per unit of frequency [42]. Integrating the PSD over a range of frequencies results in the total power in the signal for the respective frequency range. The spectral analysis was performed with the velocity signals, for which the signal to noise (voluntary to tremor) ratio was more substantial than in the position signal. The relative power change ratio, between two signals, was calculated for the tremor and voluntary frequencies. For the tremor frequencies, the power change ratio was defined between the TSO and MO as follows:

2 $$ {PC}_t=\frac{P_{tTSO}-{P}_{tMO}}{P_{tMO}}\times 100 $$

where *P*_*tTSO*_ and *P*_*tMO*_ are the total power in the TSO and MO signals for the tremor frequencies. For the voluntary frequencies, power change ratios were defined for each device relative to the target signal as follows:

3 $$ {PC}_{vTSO}=\frac{P_{vTSO}-{P}_{vTarget}}{P_{vTarget}}\times 100 $$

4 $$ {PC}_{vMO}=\frac{P_{vMO}-{P}_{vTarget}}{P_{vTarget}}\times 100 $$

where *P*_*vTSO*_, *P*_*vMO*_ and *P*_*vTarget*_ are the total power in the TSO, MO and target velocity signals in the voluntary frequencies. Voluntary motions are typically considered to have a frequency spectra below 2 Hz [43, 44], as such, the 0–2 Hz frequency range was selected in calculating *PC*_*vTSO*_ and *PC*_*vMO*_. The range for tremor includes frequencies above the voluntary [45]; to capture the power around the fundamental tremor frequency, the 2–10 Hz band was selected in this work. The PSD for the MO also yielded the participants’ fundamental tremor frequency, calculated from the PSD peak amplitude in the 3–10 Hz range.

A large attenuation percentage is desirable for the tremor signal power change, namely *PC*_*t*_. Instead, for the voluntary component a small signal power change, either positive or negative, between each device (MO and TSO) and the target signal is ideal. More importantly, the difference of the power changes (*PC*_*vTSO*_ − *PC*_*vMO*_) should be small, indicating no additional interference is introduced by the TSO.

#### Temporal analysis

In the time domain, voluntary motion tracking Root Mean Square Errors (RMSE) were calculated for the position and velocity motions between the TSO and the target and between the MO and the target. For the purpose of RMSE calculation, the TSO motion may be considered purely voluntary; instead, the MO motion contains the tremor component. By performing a zero phase LPF of the MO signal (labeled fMO) the tremor can be removed and the MO voluntary motion component can be compared to the target motion. Throughout this text, *e*_*pTSO*_, *e*_*vTSO*_ refer to the TSO position and velocity RMSE’s and *e*_*pfMO*_, *e*_*vfMO*_ to the fMO position and velocity RMSE’s, respectively. The zero phase LPF was designed empirically in Matlab using the filtfilt function and was fixed for all participants. The position signal pass and stop bands were 0.5 and 3 Hz, respectively, while the velocity signal pass and stop bands were 1.5 and 3 Hz, respectively. As in the frequency domain metric, small errors between each device and the target signal are desirable but more important is to have small differences between the devices’ position errors (*e*_*pTSO*_ − *e*_*pfMO*_) and velocity errors (*e*_*vTSO*_ − *e*_*vfMO*_). An equal amount of tracking errors with the TSO and with the fMO relative to the target would indicate no additional interference is introduced by the TSO.

Interaction torque between the user and the TSO was also measured and decomposed online using the ABPF mentioned in section Updates to control system, to obtain the voluntary component. It is desirable to have a transparent orthosis that moves smoothly with the user or, equivalently, that minimizes the interaction torque.

#### Data processing

Data from participants was recorded for at least 30 s (up to 60 s in some cases). Twenty continuous seconds were selected in order to compare continuous and representative data from all participants with both devices. Irregularities and discontinuities at data start and end, were thus limited. The procedure for selecting the specific 20 s of data time range essentially involved scanning the whole recording time in increments of 10 s, and selecting the time range with the lowest position and velocity RMSE. As an example, for a data spanning 43 s long, the position and velocity RMSE were calculated for the 10–30 s and 20–40 s time ranges. The time range with smaller RMSE values was then selected. In the sinusoid target case, the fMO and TSO signal time ranges were selected independently, which nevertheless resulted in identical target motion profile throughout the 20 s period, with no phase difference. For the pseudo-random target, 20 s of data for the TSO were selected first, following the procedure outlined above, and then the same time range was used for the fMO in order to compare identical motion profiles.

In total, four motion performance metrics were considered, one for the tremor motion (signal power change) and three for the voluntary motion (position RMSE, velocity RMSE and signal power change). The four motion cases of the pursuit task, i.e. sinusoid slow, sinusoid fast, random slow, and random fast were each performed three times (Table 1). For each participant, the three repetitions were averaged, resulting in 4 values per performance metric, representing the four motion cases. It should be noted, once selected, the same 20 s time range was used for all performance metrics in both the spectral and time domains.

#### Statistical analysis

Statistical analyses were carried out in SAS JMP for the tremor and voluntary motions. To assess the effectiveness of the approach to suppress tremor, a hypothesis was tested stating that the mean reduction in tremor with the TSO relative to the MO (*PC*_*t*_) was equal to zero suppression. For this test, a two-sided Wilcoxon Signed Rank test was used due to non-normal distribution [46], with the four protocol tasks averaged per participant. To gain an understanding of the contribution of the target motion type (i.e. sinusoid or random) and velocity (i.e. slow or fast), a two way repeated measures univariate Analysis of Variance (ANOVA) was employed. The ANOVA tested how the two independent factors, target type and target velocity, contributed to the reduction in tremor. The subject random effect was included in the ANOVA model. Post hoc analysis evaluated the suppression levels for the four conditions separately, namely sinusoid, random, slow or fast, and reported statistical differences between slow and fast, and between sinusoid and pseudo-random.

To evaluate the TSO interference to the voluntary motion, the tracking errors (*e*_*pfMO*_, *e*_*pTSO*_, *e*_*vfMO*_, *e*_*vTSO*_) and power changes (*PC*_*vMO*_ and *PC*_*vTSO*_) obtained with the MO and TSO were compared. Paired t-tests were conducted for the three performance metrics, with the four protocol tasks averaged per participant. As in the tremor analysis, the contribution of motion type and velocity on the differences between the MO and TSO performances were evaluated with a two way repeated measures ANOVA. Due to the within-subjects design in this experiment (participants’ were measured with both the TSO and MO), the error due to the human subject variability can be omitted. Thus, the dependent variables were defined as the performance metrics differences between the TSO and MO (e.g. *PC*_*vTSO*_ − *PC*_*vMO*_, for the power change). The ANOVA analysis was repeated for the three voluntary performance metrics. Post hoc analyses involved paired t-tests for the four motion profiles separately to infer their contributions to differences between the TSO and MO. Finally, correlations between the three performance metrics were evaluated to assess if they conform, namely do they similarly improve and deteriorate. A significance level of *α* = 0.05 was considered for all tests.

## Results

### Tremor motion component

**Fig. 2:**
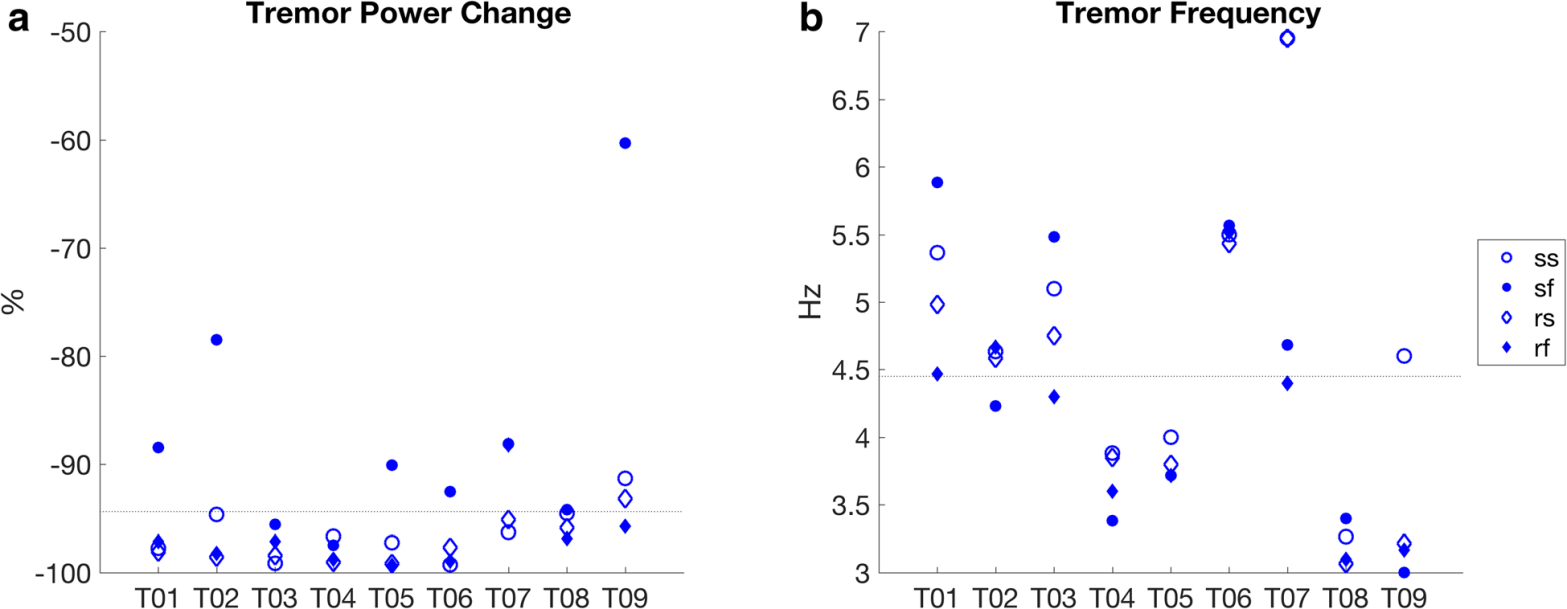
Tremor motion related measures. **a** Tremor power change and **b** MO tremor fundumental frequency (from PSD) when performing the pursuit tracking tasks. The dotted horizontal lines in **a** and **b** indicate the participants’ mean. The scatter plots are composed of four unique markers associated with the four motion cases of the pursuit tracking experiment, namely sine slow (ss), sine fast (sf), random slow (rs) and random fast (rf)

The tremor power reduction mean and standard deviation was − 94.37 (7.27)% (Fig. 2a) and was found statistically significant (*p* < 0.001) when compared to no suppression.

The ANOVA test showed statistically significant effects for the target type and target velocity interaction, as well as for the two main effects, target type and target velocity (Table 2). The ANOVA results suggest to further look at which of the four conditions, namely sinusoid, random, slow or fast, had the most tremor reduction. The tremor reductions, when considering the four conditions separately, were statistically significant (*p* < 0.001), with a mean and standard deviation of − 91.78 (9.34) for sinusoid only, − 96.96 (2.73) for random only, − 96.76 (2.28) for slow only and − 91.97 (9.57) for fast only. Additionally, better suppression is achieved for both random (*p* = 0.037) and slow (*p* = 0.015) motions. The tremor fundamental frequency mean and standard deviation was 4.45 (0.93) Hz (Fig. 2b).

**Table 2:** Pursuit tracking task summary

Sequence	Motion profile	Velocity	Repetitions	Target Velocity^a^
1	Sinusoid target	Slow	3	Sinusoid velocity: *A*_*v*_?sin(2*pft*), where *A*_*v*_?=?75.4 deg./s and *f*?=?0.3 Hz for slow case and *A*_*v*_?=?125.5 deg./s and *f*?=?0.5 Hz for fast case.
2		Fast	3	
3	Random target	Slow	3	Velocity profile was ramp shaped resulting in a parabolic position profile. Ramp top velocity: 22.2 and 50?deg./s for slow and fast cases. Ramp acceleration/ deceleration: 27.75 and 83.3?deg./s^2^ for slow and fast cases.
4		Fast	3	

All the tasks were repeated with both the MO and the TSO and recorded for approximately 30–60?s. Up to two training repetitions were offered for each device and motion profile

^a^The target parameters shown in the table are for the 0.246?cm/deg. scaling factor. A second scaling factor of 0.218?cm/deg. was also used in the study

### Voluntary motion component

No statistically significant differences were observed between the TSO and MO power change (*p* = 0.346), comparing *PC*_*vTSO*_ and *PC*_*vMO*_, or velocity errors (*p* = 0.283), comparing *e*_*vTSO*_ and *e*_*vfMO*_. The difference in position errors was, however, marginally significant (*p* = 0.05), comparing *e*_*pTSO*_ and *e*_*pfMO*_.

**Fig. 3:**
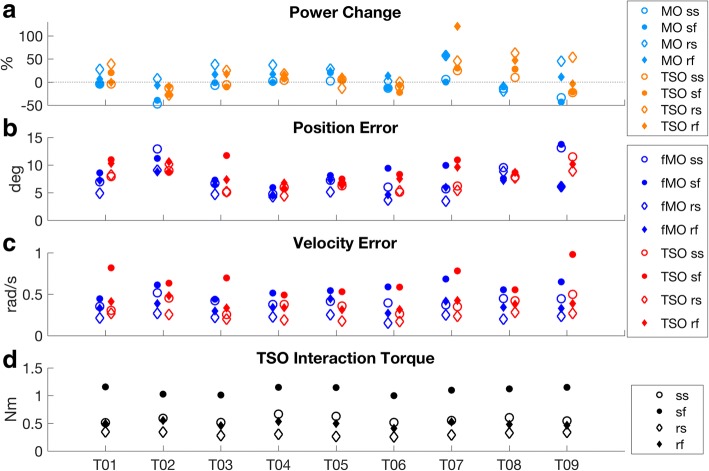
Voluntary motion related measures. **a** Voluntary component power changes for the MO (*PC*_*vMO*_) and the TSO (*PC*_*vTSO*_). The horizontal dashed line indicates zero value. **b** Position pursuit tracking RMSE for the fMO (*e*_*pfMO*_) and TSO (*e*_*pTSO*_). **c** Velocity pursuit tracking RMSE for the fMO (*e*_*vfMO*_) and TSO (*e*_*vTSO*_). **d** Voluntary interaction torque (same as Fe in Fig. 4) root mean square for the TSO. The scatter plots are composed of four unique markers associated with the four motion cases of the pursuit tracking experiment, namely sine slow (ss), sine fast (sf), random slow (rs) and random fast (rf)

The ANOVA analysis indicated the effect of the target velocity factor was statistically significant for both the position and velocity metrics, while the target type factor had an effect on the position metric only (Table 3). The above can be appreciated by observing that faster and slower motions as well as sinusoid and random motions are not evenly distributed (Fig. 3). For example, positive or larger power change values are often associated with random motions while negative or smaller values with sinusoid motions (Fig. 3 a). Conversely, larger values are repeatedly associated with sinusoid or fast motions while smaller values correspond to random or slow motions (Fig. 3b-d). Consequently, it was interesting to analyse the four motion profiles separately. For the slow motion profiles, no statistically significant differences were observed between the TSO and MO for the position (*p* = 0.586), velocity (*p* = 0.152) or power change (*p* = 0.546). For the fast motion profile, no statistically significant difference was observed for the power change (*p* = 0.269), however, both the position (*p* = 0.015) and velocity (*p* = 0.033) errors were statistically different. As for the sinusoid motion profile, no statistically significant differences were observed between the TSO and MO for the position (*p* = 0.944) or velocity (*p* = 0.311), however a difference was observed for the power change (*p* = 0.044). For the random motion profile, no statistically significant differences were observed for the velocity (*p* = 0.439) or power change (*p* = 0.825), however a difference was observed for the position (*p* = 0.003).

**Table 3:** ANOVA for tremor suppression

		Source		
		Target type	Target velocity	Target Type *Target Velocity
Tremor Power Change (%)	*F*(1,8)	6.26	9.67	5.7
	*p*	0.037	0.015	0.044

The means and standard deviations of the MO and the TSO performance metrics were 4.32 (16.37)% and 10.96 (22.91)% for the power change, 7.3 (1.9) deg. and 8.2 (1.9) deg. for the position RMSE and 0.39 (0.04) rad/s and 0.41 (0.07) rad/s for the velocity RMSE. The TSO interaction torque mean and standard deviations was 0.62 (0.04) Nm.

**Fig. 4:**
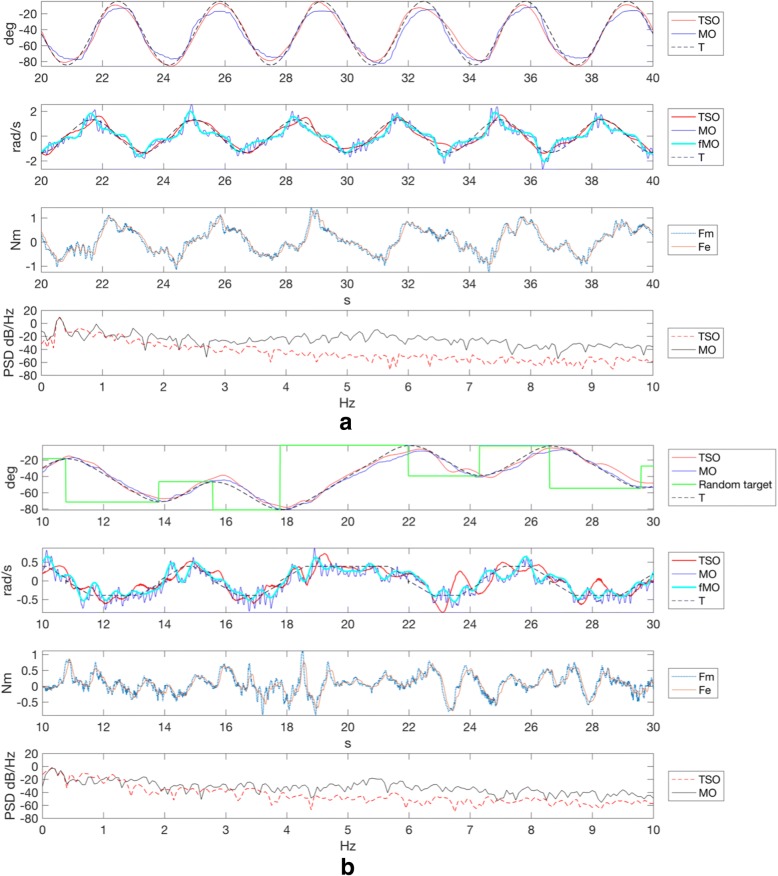
Participant T06 sine and pseudo-random target tasks. **a** Slow sinusoid target motion **b** Slow pseudo-random target motion. For both subfigures **a** and **b**, the top two subplots show the position and velocity associated to the target T, the TSO and the MO. The velocity plot also shows the fMO velocity. The pseudo-random position plot also shows the successive random targets (solid green square wave). The bottom two subplots refer to the TSO interaction torque and the PSD obtained for both the TSO and MO. The measured torque Fm represents the combined voluntary and tremor components. The estimated torque Fe represents the voluntary component as a result of online filtering

Representative tracking and PSD plots for participant T06, corresponding to the slow sinusoid and slow random target motion profiles, are shown (Fig. 4). It can be observed in the interaction torque subplots that Fm contains a high frequency component (tremor motion), which is filtered in Fe, representing the voluntary component. In the PSD plots, it can be observed the TSO and MO curves overlap closely around the voluntary motion frequency (~ 0.5 Hz). Instead, in the higher frequencies of the tremor (~ 5–6 Hz) the TSO magnitude is appreciably reduced.

**Fig. 5:**
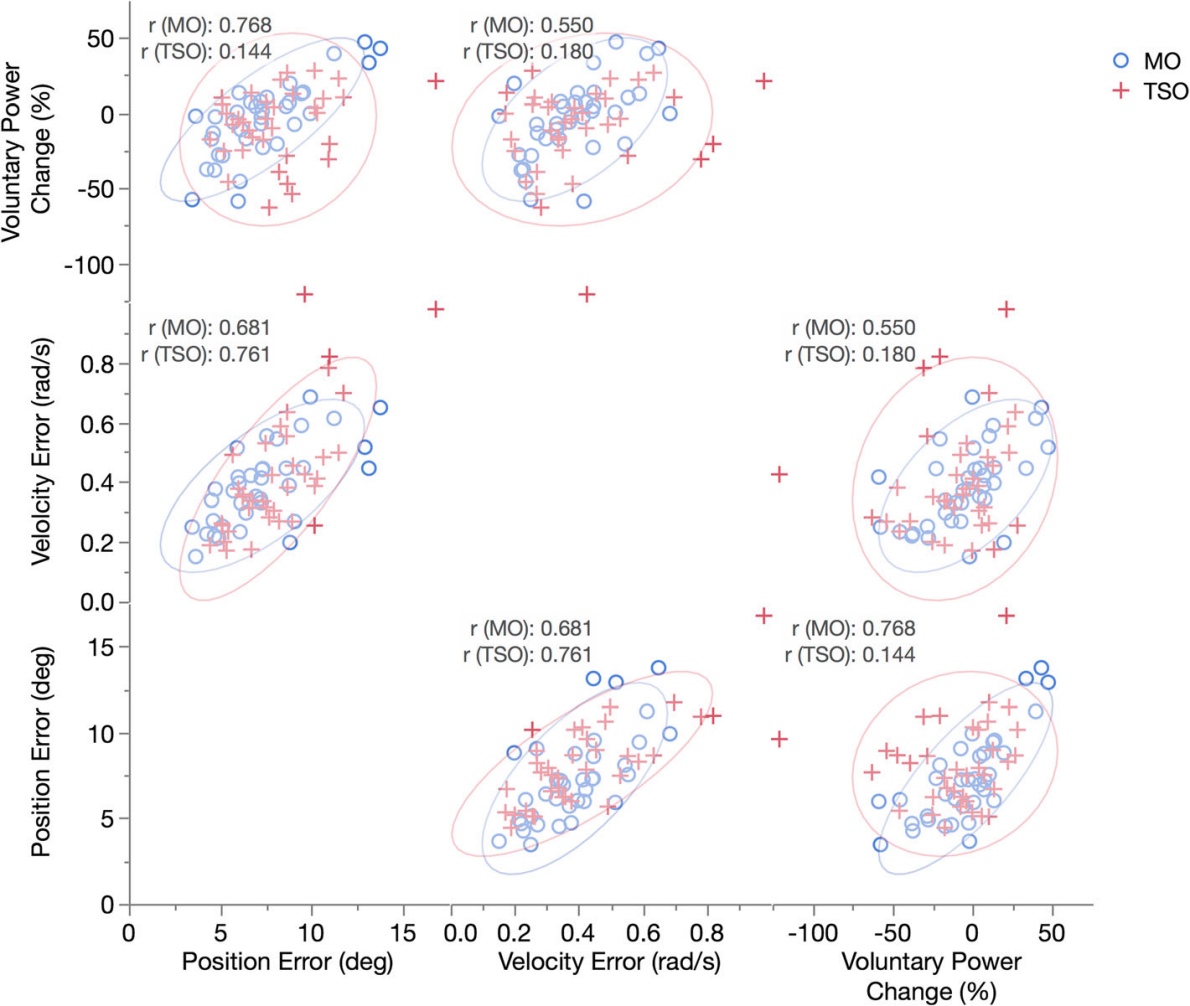
Performance Metrics Correlations Matrix. Each correlation is composed of 36 data points (nine participants performing the four motion cases)

A correlation analysis of the three voluntary motion performance metrics confirms that when there is an increase in interference due to TSO, it affects position similarly to velocity and to a lesser extent to power change (Fig. 5). Narrow ellipses indicate a stronger correlation. It is evident there is a greater variability with more outliers for the TSO resulting in weaker correlations for the power change vs. position RMSE (*p* = 0.14) and voluntary power change vs. velocity RMSE (*p* = 0.18). All other correlations were statistically significant (*p* < 0.01).

## Discussion

This tremor suppression investigation relies on a recently developed suppression approach coupled with a previously tested wearable technology targeting the elbow joint [37], which has been shown to be central to most activities of daily living [47, 48]. In evaluating the suppression approach, the effects to both involuntary and voluntary motions are considered. The results indicate better tremor suppression than comparable interventions. Moreover, voluntary motion interference is shown to be limited, statistically speaking, albeit not completely eliminated.

All participants in this study were right-handed. For all participants, other than T01, T07 and T08, the tremor was more severe on the right side and all but one participant (T08) were tested on the more severe side. Due to a medical condition, T08 was tested on the right side (left side was more severe). It is interesting to note that in the literature there is conflicting evidence as to whether the more severe side is likely to be the dominant side [49], the non-dominant side [50, 51] or neither [52].

Two key observations related to the tremor measures that can be appreciated visually in Fig. 2a are that attenuation was overall higher for random relative to sinusoid target movements and for slower relative to faster target movements. A matching subjective observation of the participants was that tracking faster target motions was easier to perform while inducing less tremor. There was a relatively large variation between ET participants mean frequency in Fig. 2b (T01, T04, T05, T06, T08 and T09). Only two of the recruited participants were identified as PD (T02 and T03); therefore, it is difficult to determine if similar frequency variations would be observed among the PD subjects. The typical bandwidth for ET, however, is considered to be wider, overlapping below and above that of PD [1]. The intrasubject tremor frequency variations in this study are comparable with those in [53] but smaller than in [54]. In instances were a clear tremor peak was not detectable (mainly for T08 and T09), the PSD frequency peak search would occasionally result in a frequency near the search lower limit of 3 Hz. The TETRAS score was fairly consistent across study participants as evidenced by the small standard deviation in Table 4. For participant T09 who had substantially milder tremor, which was barely visible, it is interesting to note also a substantially lower tremor reduction of 85%.

**Table 4:** ANOVA for voluntary performance metrics differences

Source	Position RMSE (deg) (*e*_*pTSO*_ − *e*_*pfMO*_)		Velocity RMSE (rad/s) (*e*_*vTSO*_ − *e*_*vfMO*_)		Voluntary Power Change (%) (*PC*_*vTSO*_ − *PC*_*vMO*_)	
	*F*_1,32_	*p*	*F*_1,32_	*p*	*F*_1,32_	*p*
Target Type	9.87	0.0036	0.31	0.58	1.03	0.32
Target Velocity	4.96	0.033	8.61	0.0061	0.29	0.59
Target Type *Target Velocity	0.62	0.44	5.04	0.032	0.24	0.63

Observations related to the voluntary measures are considered next. Random motion tasks were associated with larger power change values and lower tracking errors as can be appreciated from Fig. 3. Slow motions were also associated with lower tracking errors. The above is likely related to the lower speed and the more gradual increase in motion range of the random tasks. Despite the two weaker correlations in Fig. 5 for the TSO, generally, an improvement in one metric suggests improvements of other metrics. Of key interest in this study is the difference in the voluntary performance of the TSO relative to the MO. A velocity controller was utilized in the suppression approach which may explain the paired difference test marginal result for position RMSE but not for velocity RMSE or voluntary power change, which was also based off the velocity signal. Participant T08 demonstrated the largest difference in the voluntary power change metric (49.88%) between the MO and the TSO (Fig. 3a). The large voluntary power change difference may be related to the limited ROM the participant was achieving with the MO compared to the TSO. The difference in ROM could be due to device fitting issues, or alternatively, to the TSO inherent actuation enabling greater ROM (more signal power). Corresponding large differences do not appear in the subject’s position or velocity RMSE’s, in Fig. 3b and c. For participant T08, only two repetitions for the fast random movement were collected. Beyond paired difference tests, it is important to consider the effect sizes for the differences between the TSO and MO were only 0.9 deg. for the position RMSE, 0.02 rad/s for the velocity RMSE and 6.64% for the power change (section Voluntary motion component). Furthermore, when excluding T08, the power change between MO and TSO increases by only 1.23%. Referring to Fig. 3d, tasks with an overall lower velocity resulted in overall lower interaction torque, as expected from an admittance controlled system.

Potential limitations are recognized, related to the suppression system and approach. Overall, high velocity motions have been shown to have a dominant contribution to degraded voluntary performance. Also, voluntary interaction forces should be further reduced in future work. Some looseness and play between the orthosis and the human arm as well as within the orthosis mechanism exists, potentially resulting in some of the tremor not being detected. Modifications to reduce backlash and play may increase the signal to noise ratio and thus improve the tremor suppression. Follow up explorations should also address limitations to the study design. The study does not evaluate the passive effect of the mass and inertia of the TSO on tremor suppression and on voluntary motion. Thus, in future work the suppression with the TSO in off mode should be demonstrated to assess the mass and inertia contribution. Whether the tremor suppression is primarily caused by the active suppression approach or the passive mass and inertia is, however, deemed less critical so long that the negative impact to the voluntary motion remains limited, as shown in this work. Furthermore, the suppression approach demonstrated significant attenuation using substantially less mass and inertia in previous work [36]. Another potential protocol limitation involves order effects due to fatigue and training when the TSO is tested after the MO. If these effects exist, they may be mitigated by randomizing the TSO and MO order. Fatigue may have also occurred due to the TSO resistance, particularly at later stages in the testing session, and may have worsened voluntary tracking results. TETRAS was used to score two PD subjects in this study despite being targeted at ET. Additionally, TETRAS scoring may have been influenced by the preceding device testing. Nevertheless, if there was an effect due to the device testing, it may be assumed to be roughly equal for all participants. Since this is an assistive device, the TETRAS outcome is not considered crucial for the device’s performance evaluation. Therefore, in this preliminary study, the implication of post testing assessment as well as PD subjects assessment is not considered critical. The study protocol evaluated tremor during active movement. In future work, rest tremor could be incorporated for evaluation. RMSE position and velocity measures could potentially also be used to assess tremor suppression. In this work, the voluntary component contribution to tracking RMSE was much more substantial relative to the tremor contribution, perhaps due to the large voluntary motion amplitude or due to sensor sensitivity. As a result, comparing the MO to the TSO tracking errors resulted in poor RMSE tremor suppression sensitivity. Subjects were asked to abstain from medications for 12 h before testing, yet this time span may not be enough for ET subjects (taking Primidone or Propranolol) to be in a clinically-defined off state. Although tremor was observable in all study subjects, by motion sensors and by TETRAS, the implication is that tremor could be more severe in fully off-medication individuals. It is expected, however, that an increase in tremor signal would result in better voluntary and tremor decomposition, by the control system, and consequently better voluntary motion tracking and noise (tremor) rejection. In future work, recording of medications may help to guide abstinence times. Patients with PD may exhibit involuntary and burst-like movements, other than tremor, such as dyskinesia, with a frequency spectra overlapping both voluntary and tremor motions. It should be noted, our inclusion criteria did not explicitly identify such movement abnormalities. The suppression approach is designed such that motions residing within the frequency band defined for tremor (2–10 Hz) are considered a disturbance and consequently rejected. Our device and control approach successfully rejected motions in this frequency band during the tests as demonstrated in Figs. 2 and 4 (PSD plots). Non-tremor motor disorder movements that overlap tremor frequencies are therefore suppressed along with the tremor. On the other hand, we expect that our system will not be effective in suppressing involuntary movement in the frequency band 0–2 Hz and that voluntary motions will be affected by such motor disorders. Nevertheless, the effect to the MO and TSO voluntary performance may be roughly the same such that voluntary tracking can still be compared and evaluated.

The MO was used in this study mainly as a performance benchmark for the TSO. Nevertheless, in a real world scenario, per user initialization of the ABPF center frequency would be needed and could still be performed with the MO, as was done in this study. Alternatively, initialization and if needed, update of the tremor center frequency may be realized without the MO by modifying the TSO and the tremor estimation algorithm. A few participants commented they noticed a favourable effect of the suppression on their tremor and some expressed interest in such a device if size and weight were reduced. Other studies reported some migration of tremor to nearby joints [29]. A similar phenomenon was not observed in this study. It is expected that by adapting the suppression orthosis to other joints (e.g. the wrist), a similar alleviation of tremor would be observed. However, follow-up investigations would be needed to verify this. Additional studies, involving larger populations, are also needed to validate the technology. The ABPF fundamental tremor frequency was the only subject related parameter requiring calibration in the proposed system, reducing the approach sensitivity to different users or neurophysiological changes.

## Conclusion

This preliminary study aims to demonstrate the feasibility of a recently developed tremor suppression method. Participants with varying tremor severities were recruited and benefited from a 94.4% tremor reduction. Moreover, the suppression device restriction to the voluntary motion was explicitly addressed and quantified, different from similar studies involving individuals with tremor. Specifically, the mean voluntary position and velocity tracking errors increased by only about 1 deg. and 0.02 rad/s, respectively when using the TSO, while the voluntary signal power change increased by 6.6%. Overall, a marginally statistically significant effect of the TSO was observed only with regard to the voluntary position error. The demonstrated results for both the tremor and voluntary motions suggest the tremor suppression approach can be beneficial for people affected by tremor.

## Abbreviations

DBS: Deep brain stimulation
ET: Essential tremor
FES: Functional electrical stimulation
fMO: filtered MO
LPF: Low pass filtering
MO: Measurement orthosis
PD: Parkinson’s disease
PSD: Power spectral density
RMSE: Root mean square error
ROM: Range Of Motion
TETRAS: The essential tremor rating assessment scale
TSO: Tremor suppression orthosis
